# Loop Ileostomy in Europe's Tiniest Male Newborn for Meconium-Related Ileus

**DOI:** 10.1055/s-0040-1721406

**Published:** 2021-03-03

**Authors:** Holger Till, Georg Singer, Christoph Castellani, Berndt Urlesberger

**Affiliations:** 1 Department of Paediatric and Adolescent Surgery, Medical University of Graz, Graz, Austria; 2 Department of Paediatrics and Adolescent Medicine, Division of Neonatology, Medical University of Graz, Graz, Austria

**Keywords:** laparotomy, loop ileostomy, periviable fetus, meconium-related ileus, extremely low birth weight

## Abstract

With the advances of neonatology, the survival rate for “live-born periviable fetuses” weighing < 300 g, a subgroup of extremely low birth weight (BW) infants, has improved over the past 10 years. Meconium-related ileus (MRI) represents an early postnatal hazard, and, if medical evacuation fails, a surgical challenge in such immature babies. We report the interdisciplinary management of surgically treated MRI in a newborn with a BW of 273 g. According to the worldwide database held by the University of Iowa, he is registered as the tiniest male newborn in Europe. The boy was born in the 25th gestational week by cesarean section after a triplet pregnancy with twin–twin transfusion syndrome, him being the donor. He had a BW of 273 g, whereas his brothers had a BW of 740 g and 722 g. Cardiopulmonary stabilization and ventilation were successful. He developed MRI unresponsive to medical treatment. On day 14 of life, a minilaparotomy was performed in the right lower quadrant to externalize a loop of the distal ileum in a no-touch technique. Despite the small diameter of only 2 mm, a standard loop ileostomy could be fashioned. There were no intra- or postoperative abdominal complications. Bowel function and weight gain were adequate and the ileostomy was closed electively 5 months later at a body weight of 3.5 kg.

In summary, minilaparotomy and loop ileostomy placement were effective to treat surgical MRI in Europe's tiniest male newborn. With the advances of neonatology, pediatric surgery reaches new frontiers as well.

## Introduction

The term meconium-related ileus (MRI) was first introduced by Kubota et al in 1999 1 and has been generally accepted today 2 summarizing all meconium-related obstructions apart from cystic fibrosis. Kubota et al argued that the meconium plug syndrome of the colon and the meconium disease of the distal ileum have similar origins for an ineffective peristalsis of the sticky adherent meconium due to an immaturity of the enteric nervous system, especially the pacemaker cells of Cajal and ganglia. 1 2

Medical evacuation remains the first-line treatment. 3 4 However, if it fails and increased intraluminal pressure develops, MRI becomes a surgical challenge to avoid bacterial translocation, sepsis, perforation, or even death. 2 5 6

We report the interdisciplinary management of a newborn with a birth weight (BW) of only 273 g.

## Case Report

The neonate was born in the 25th + 3 gestational week by cesarean section after a triplet pregnancy with twin–twin transfusion syndrome, him being the donor. He had a BW of 273 g and a body length of 26 cm, whereas his brothers had BWs of 740 g and 722 g. According to the database of the world's “Tiniest Babies,” held by the University of Iowa, he is registered as the tiniest male newborn in Europe ( https://webapps1.healthcare.uiowa.edu/TiniestBabies/getInfantList.aspx ).

**Fig. 1:**
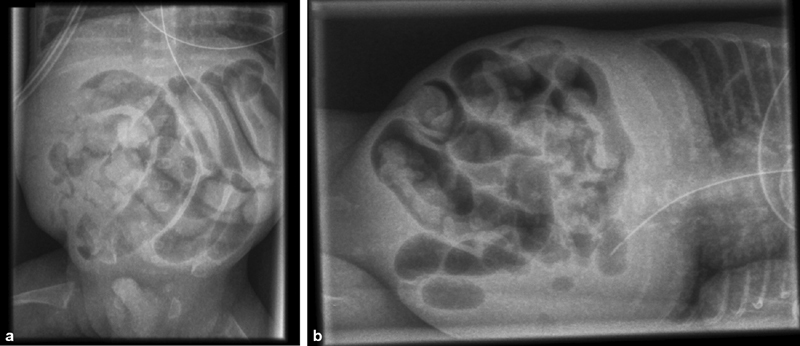
X-rays on day 14 of life in supine ( **a** ) and left lateral ( **b** ) positions revealed distended gas- and meconium-filled small intestinal loops; no intra-abdominal free air, air-fluid levels, or pneumatosis was noted.

The infant showed respiratory distress syndrome grade 2 requiring immediate intubation and application of surfactant (200 mg/kg). Extubation could be performed on day 7. Gradually, he developed MRI unresponsive to medical treatment consisting of daily enemas with diluted Microlax. Additionally, the patient received two enemas with diluted water-soluble contrast media (Gastrografin). Abdominal X-rays on day 14 of life revealed distended meconium- and gas-filled small intestinal loops ( Fig. 1 ). Abdominal sonography showed distended intestinal loops without bowel wall thickening or pneumatosis, confirming MRI. Subsequently, the clinical situation of the patient deteriorated with increasing abdominal distention and cardiorespiratory instability. Therefore, a minilaparotomy (1 cm) was performed in the right lower quadrant to externalize a loop of the distal ileum in a no-touch technique. Despite the small diameter of only 2 mm, a standard loop ileostomy could be fashioned. There were no intra- or postoperative abdominal complications, explicitly no liver hemorrhage, no prolapse of the ileostomy, and no revisional surgery later on. Bowel function and weight gain were adequate, and the ileostomy was closed electively 5 months later at a body weight of 3.5 kg.

## Discussion

Although the survival rate of extremely low BW (ELBW) infants with a BW less than 500 g has significantly improved over the last 5 to 10 years, 7 8 9 the incidence of major morbidities determining their long-term prognosis remains rather unchanged. Besides cardiopulmonary and neurological sequelae, abdominal complications such as MRI represents an early postnatal hazard.

Obstruction of the gastrointestinal tract by tenacious meconium frequently leads to gastric residuals, a distended abdomen, and delayed food passage. Even in such extremely small babies, rapid evacuation using prophylactic enemas plays a key role to prevent MRI. 3 Any delay in the initiation of medical therapy could increase the risk of emergency surgical interventions. 4

If medical treatment fails and clinical deterioration develops, surgical decompression must be initiated despite the immaturity of the infant 4 to avoid bacterial translocation, sepsis, or even potentially lethal perforation. According to Byun et al, male gender and ELBW are independent risk factors for developing surgical MRI. 2 Once uncorrectable thrombocytopenia, acidosis, and cardiopulmonary insufficiency associated with prolonged MRI occur, the prognosis worsens considerably.

Abdominal surgery in ELBW infants, especially in babies weighing less than 500 g, carries an increased risk for higher postoperative morbidity and mortality versus very low BW infants. 6 Major factors are attributable to the fragility of the intestinal tissue and vulnerability of the hepatic capsule to tearing on manipulation. 5 Consequently, in our ELBW infant, the minilaparotomy was deliberately placed in the right lower quadrant to avoid any interference with the liver. It allowed identification of the terminal ileum, which was not pulled out but exteriorized by gently pushing on the abdominal wall without touching the tissue. Technically, our loop ileostomy placement for MRI was not much different from other abdominal diagnoses such as necrotizing enterocolitis (NEC). Few delicate interrupted sutures were sufficient to fix the bowel to the fascia and allow for permanent function without reoperation.

Generally, the outcome after surgery for MRI in ELBW infants has improved significantly from early mortality rates of 50% to 67% to present survival rate of near or at 100%. 5 10

In detail, Kim et al 6 found in their study that there were no cases of recurrent MRI after surgery and none of the infants developed NEC after feeding. Consequently, enteral feeding patterns after surgical procedure were not significantly different based on BW in the immediate postoperative period.

In our case, postoperative feeding followed the neonatal standards. 11 There were no recurrent surgical MRI or revisional surgery, but there was adequate growth pattern. Bowel function and weight gain were adequate, and the ileostomy was closed electively 5 months later at a body weight of 3.5 kg.

In summary, we report a successful interdisciplinary management of MRI in Europe's tiniest male newborn (BW of 273 g). His favorable outcome following surgical MRI substantiates the debate that delicate, interdisciplinary management, precise timing, and minimal handling seem key factors of long-term survival with adequate quality of life in such extremely low BW babies.

## Notes

New Insights and the Importance for the Pediatric Surgeon We report the successful, interdisciplinary management of a meconium-related ileus (MRI) in Europe's tiniest male newborn (birth weight of 273 g). His favorable outcome following surgical MRI substantiates the debate that delicate, interdisciplinary management, precise timing, and minimal handling seem key factors of long-term survival with adequate quality of life in such extreme babies.
